# People and Plants in Nunatsiavut (Labrador, Canada): Examining Plants as a Foundational Aspect of Culture in the Subarctic

**DOI:** 10.1007/s12231-021-09530-7

**Published:** 2021-11-01

**Authors:** C. H. Norton, A. Cuerrier, L. Hermanutz

**Affiliations:** 1 Institut de Recherche en Biologie Végétale de L’Université de Montréal, Montreal, Canada; 2 Department of Biology, Memorial University of Newfoundland, St. John’s, Newfoundland and Labrador, Canada

**Keywords:** Nunatsiavut, Labrador, Northern communities, Inuit, Eastern Subarctic, Berries, Ethnobotany

## Abstract

This paper emphasizes the cultural value of plants in Nunatsiavut (Labrador, Canada), a self–governing Inuit region in the Subarctic. Via interviews with community members, we describe the links between plant usage and culture to understand the direct ways that plants are utilized for food, construction, gardening, and medicine, and to then link these uses to deeper cultural significance among three communities in Nunatsiavut (Hopedale, Postville, and Rigolet). Many plants were common amongst communities with a total of 66 taxa identified. About 75% of taxa were reported in at least two communities, corresponding to 95% of all responses. Edible plants were the most common reported usage, with emphasis on berry–producing taxa such as blueberry shrubs. Our study shows that a diversity of plants (i) support cultural activities; (ii) act as markers for historical events; (iii) highlight intergenerational exchange and valuing of plant knowledge; (iv) express the deep awareness that people have for their local environment; and (v) a medium for the expression of traditional values. The similarities in the plant responses among the communities suggest a shared body of plant knowledge. Our study supports the great cultural importance of plants in northern communities.

**Supplementary Information:**

The online version contains supplementary material available at 10.1007/s12231-021-09530-7.

## Introduction

Nunatsiavut is a self–governing Inuit territory located along the northern coast of Labrador, in the province of Newfoundland and Labrador, Canada. Nunatsiavut means “Our beautiful land” in the Nunatsiavut dialect of Eastern Canadian Inuktitut (Dorais 2010). The region spans a number of ecoregions, including Coastal Barrens, High Subarctic Tundra, and Mid–Subarctic Forests, among others (Roberts et al. 2006). Ancestors of Inuit have lived in the region for millennia (Brice-Bennett et al. 1977).

There is now firm recognition of plants’ importance to northern communities, but this was not always the case (Oberndorfer et al. 2017). Looking specifically at Inuit plant usage, Norton (2019) compiled nearly a hundred texts describing plant usage in communities from northern Alaska, the Canadian Western Arctic, the Canadian Eastern Arctic and Subarctic, and Greenland. Norton (2019) collated a total of 311 plant taxa, corresponding to 73 taxonomic families, that were noted as serving a purpose in Inuit activities and culture. We can confidently reject outdated, reductionist views that described plants as just a source of vitamin C, a negligible source of calories, and not much else in the Arctic and Subarctic (Boas 1888; Hoffmann et al. 1967; Porsild 1953; Rodahl 1952).

Today, there are five vibrant communities in Nunatsiavut, all of which are coastal. The communities, going North to South, are Nain, Hopedale, Makkovik, Postville, and Rigolet. We did ethnobotanical interviews with community members in Hopedale, Postville, and Rigolet. Nain and Makkovik have recently participated in in–depth ethnobotanical surveys (Clark 2012 and Oberndorfer 2016, respectively). Clark (2012) compared plant use between Nain and another Inuit community in Nunavik. Oberndorfer (2016) focused on the links between plants and people and culture in Makkovik. This paper describes more than one community, like Clark (2012), but with greater focus on teasing apart the intricate ways that plants act as a means for the expression of local culture, as in Oberndorfer (2016). The two main goals of this research are to (a) understand how plants are used in Hopedale, Postville, and Rigolet; and (b) tease apart the deeper, more fundamental ways that plants support present day culture in Nunatsiavut.

## Methods

### Study Area

**Fig. 1:**
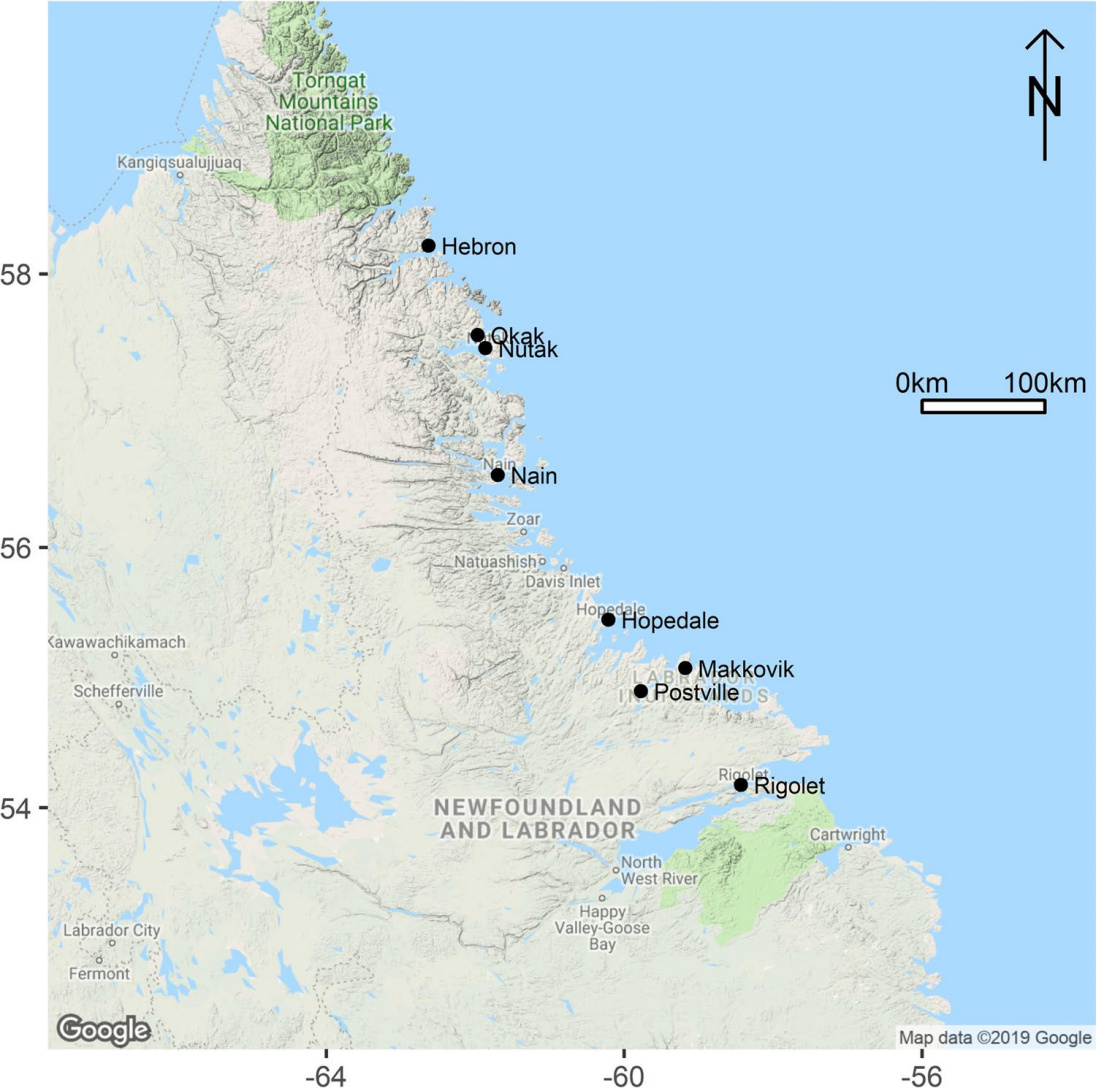
Map showing the locations of communities in Nunatsiavut, on the northeastern coast of the Labrador Peninsula facing the Labrador Sea, made using the ggmap package in R Studio. The three relocated communities are also shown north of Nain: Nutak, Okak, and Hebron. See the bottom right corner of the map for attribution.

Nunatsiavut is one of four Inuit regions in northern Canada, the other three being the Inuvialuit Settlement Region (Yukon and Northwest Territories), Nunavut, and Nunavik (Northern Quebec). Nunatsiavut is mostly Subarctic, including the three communities in this study, but the most northern tip of the territory is Arctic (Roberts et al. 2006). Hopedale, Postville, and Rigolet are three of the five communities that make up Nunatsiavut (Fig. 1).

As of the federal census in 2016, Hopedale had a population of 574, Postville, 177, and Rigolet, 305 (Statistics Canada 2016a, 2016b, 2016c). The Nunatsiavut population is a mix of Inuit and settlers of European heritage (Brice-Bennett et al. 1977).

Three historical events permeate culture in Nunatsiavut. First, Moravian missionaries—a German protestant denomination—began establishing missions in this region in the mid–eighteenth century, all of which ended in the late twentieth century. Second, the Spanish Flu epidemic in the early twentieth century ravaged northern Labrador, even forcing the closure and resettlement of Okak, a community north of where Nain is today (Fig. 1). Third, the provincial government of Newfoundland and Labrador implemented forced relocations in the late 1950s of Inuit from Nutak and Hebron, both of which were located further north than existing communities today. Inuit living in these communities were moved to the more southern communities of Nain, Hopedale, and Makkovik, and this history informs their knowledge of plants.

### Data Collection

Our team has been carrying out ethnobotanical research in partnership with Nunatsiavut communities for over 10 years and have a strong bond with the Nunatsiavut Government research unit and the communities. We returned all recordings of interviews, transcripts, photos, and outputs (e.g., scientific posters, theses, etc.) to the Nunatsiavut Government, as per our research permit. We engaged a community co–author, but due to work commitments, they were ultimately unable to participate. We held two community input sessions to help shape the research in the interest of the communities. To ensure outcomes were repatriated to the communities after the study was completed, we returned to the communities and held two informal plant–workshops to share what we had learned.

We want to note that this paper is written from the perspective of “us” (i.e., Settler researchers) documenting “their” (i.e., community members in Nunatsiavut) plant usage. We never mean to imply that plant usage only becomes valid when it is recorded by Settler researchers like ourselves. Nor do we purport that we have documented an exhaustive list of culturally significant plants in Hopedale, Postville, and Rigolet. Put simply, our research is one snapshot in time of this dynamic system of plants and culture in Nunatsiavut. Should there ever be future discrepancies between our findings and what communities say, the deference must always be given to the communities.

Data collection consisted of semi–structured interviews with mature community members over the age of 18. Interviews consisted of questions about how plants were used for eating, medicine, and crafting, etc. In the context of these interviews, a “plant” was defined in colloquial terms and included a wide range of nonvascular (lichens and algae) and vascular plants as well as plant affiliates.

Interviews did not follow a strict questionnaire format, but instead explored topics based on the people’s interests. Generally, most interviews began with questions about berry picking, and then led into topics such as smoking fish, medicinal plants use, wood burning, and liked/disliked plants. Interview locations were determined by the interviewee and took place in homes, offices, and public spaces. We found people to interview based on recommendations from other members in the community, in addition to paper and online advertisements. Interviews were conducted in English, but there were three interviews during which an interpreter helped translate between Inuttut and English. Interviews in Hopedale and Postville took place in June 2017 by CHN. Interviews in Rigolet were conducted in March 2015 by AC and Vanessa Mardones, then a student in the lab group researching a specific medicinal plant (Mardones 2019).

Plants were classified using VASCAN (data.canadensys.net/vascan), the Digital Flora of Newfoundland and Labrador (digitalnaturalhistory.com/flora.html), and The Plant List (http://www.theplantlist.org), in addition to previous plant use surveys in Nunatsiavut (Clark 2012; Oberndorfer 2016) and local field guides (Cuerrier and Hermanutz 2012; Downing et al. 2012). We tried to identify responses to the lowest level of taxonomic classification considered for this survey, the species level. Such specificity was not always possible due to a lack of one–to–one correspondence with common names. For example, a person may report “redberry,” and this was easily associated with *Vaccinium vitis–idaea*. However, if a person discussed a “willow,” it was not possible to classify below the genus *Salix*.

### Data Handling and Processing

We audio–recorded and then transcribed most interviews. Detailed notes were taken for those interviewees who were not comfortable being recorded. Each plant mentioned in each interview was collated in a table, recording how each plant was used, in addition to plant family, local common name, plant functional group (tree, shrub, herb, etc.), and which part of the plant was used for what purpose. We sorted plant usage into nine categories, as per Clark (2012): edible, medicinal, fire, design, garden, game, avoid, decoration, and miscellaneous. We imported this spreadsheet into R Studio for tabulations (see Electronic Supplementary Material [ESM] 1). We made the map of Nunatsiavut in R Studio using the ggmap package. We made our bar plot in MSExcel and our tables in MSWord.

## Results

### Demographics of People Interviewed

In total, there were 30 interviews, which included 32 interviewees. We conducted eight interviews in Postville (seven women; one man), fifteen in Hopedale (eleven women; six men), and seven in Rigolet (four women; three men). We interviewed 22 women and 10 men in total. While we recognize that gender influences plant knowledge (Ayantunde et al. 2008 and Voeks 2007, as examples), we are interested in plant use broadly, and hence did not consider it within the scope of this paper. The average age of participants was approximately 64 years, and ranged from 48 to 90 years old.

### Taxonomy, Plant Group, Frequency, and Usage among Communities

There was a total of 61 taxa and five broad categories (rotten wood, seaweed, wood, brush, and tree) that did not relate to any taxonomic grouping reported. For simplicity, these will all be henceforth referred to as “taxa,” resulting in a total of 66 taxa. Of the 66 taxa, over half (34) were reported in all three communities (Table 1). Additionally, 15 taxa were common to two of the three communities (Table 2).

**Table 1:** Table of the 34 taxa reported in Postville, Hopedale, and Rigolet*.

Reported common name(s)	Most specific classification	Family	Plant Group	Use(s)	Frequency
Blueberry, ground hurts, tobacco hurts	*Vaccinium* spp*.*	Ericaceae	Shrub	Edible	30
Blackberry, crowberry	*Empetrum nigrum* L	Ericaceae	Shrub	Edible, fire, design, miscellaneous (pest repellent, partridge food)	29
Bakeapple, cloudberry	*Rubus chamaemorus* Fisch. ex Ser	Rosaceae	Herb	Edible	29
Redberry, partridgeberry	*Vaccinium vitis–idaea* L	Ericaceae	Shrub	Edible, medicinal, fire, design	28
Labrador tea, Indian tea	*Rhododendron groenlandicum* Kron & Judd	Ericaceae	Shrub	Edible, medicinal, fire, design, avoid	20
Rhubarb	*Rheum compactum* L	Polygonaceae	Herb	Edible, garden	18
Willow, low willow	*Salix* spp*.*	Salicaceae	Shrub	Edible, medicine, design, avoid, decorate, miscellaneous (pest repellent, animal food, indicator for water)	18
Spruce	*Picea* spp*.*	Pinaceae	Tree	Edible, medicinal, fire, design, game, decorate, miscellaneous (partridge food)	17
Foxberry, bearberry, dog berry	*Arctous alpina* (L.) Nied	Ericaceae	Shrub	Avoid	16
Tulligunuk, tunialuk, two–lee –oo–nuck	*Rhodiola rosea* L	Crassulaceae	Herb	Edible, medicinal, garden, game, decorate, miscellaneous (food for gulls)	16
Birch	*Betula* spp*.*	Betulaceae	Tree	Edible, fire, design, garden, avoid	15
Juniper	*Larix laricina* (Du Roi) K.Koch	Pinaceae	Tree	Edible, medicinal, fire, design, garden, avoid, decorate, miscellaneous (as toilet paper)	15
–	Wood	–	Tree	Edible, fire, design, game	14
Vir, fir	*Abies balsamea* (L.) Mill	Pinaceae	Tree	Edible, medicinal, fire, design, garden, decorate, miscellaneous (for puppy beds)	13
Dogberry, dogwood tree	*Sorbus decora* (Sarg.) C.K.Schneid	Rosaceae	Tree	Edible, medicinal, garden, decorate, miscellaneous (indicator for potential snowfall)	12
Raspberry	*Rubus idaeus* L	Rosaceae	Shrub	Edible	11
Shark’s blanket, flat seaweed, kellup, kelp	Order Laminariales	–	Alga	Edible, garden, avoid	10
Seaweed with bubbles, kellup, rockweed	Class Phaeophyceae	–	Alga	Edible, garden, game	10
Currant	*Ribes glandulosum* Grauer	Grossulariaceae	Shrub	Edible	10
Squashberry	*Viburnum edule* Raf	Adoxaceae	Shrub	Edible	10
Rotten wood	Rotten wood	–	Fungus	Edible, medicine, fire	9
Chives, wild chive, wild onion	*Allium schoenoprasum* L	Amaryllidaceae	Herb	Edible, garden	8
Mushroom, puffball	Division Basidiomycota	–	Fungus	Edible, medicinal, design, avoid	8
Salt grass, saltwater grass, tidal grass, grass, lime grass, sewing grass	*Leymus mollis* (Trin.) Pilg	Poaceae	Herb	Edible, design	8
Kellup	Seaweed	–	Alga	Garden	8
Hemlock	*Angelica atropurpurea* L	Apiaceae	Herb	Fire, game, avoid	7
Dandelion	*Taraxacum* spp*.*	Asteraceae	Herb	Edible, avoid, decorate	7
Fireweed, salmon flower, bumblebee flower	*Chamaenerion angustifolium* (L.) Schur	Onagraceae	Herb	Game, avoid, decorate, miscellaneous (bloom indicates that salmon are coming)	6
Poison ivy, vetch, Jacob’s ladder	*Vicia cracca* L	Fabaceae	Herb	Avoid	5
Crackerberry, crackers	*Cornus* spp*.*	Cornaceae	Herb	Edible, game, avoid	5
Aspen, poplar, asp	*Populus balsamifera* L	Salicaceae	Tree	Fire, design, garden, avoid	5
Strawberry, raspberry, beach strawberry, wild strawberries	*Rubus arcticus* L	Rosaceae	Shrub	Edible	5
Cottongrass, puffin plant	*Eriophorum* spp*.*	Cyperaceae	Herb	Medicinal, fire, design, decorate, miscellaneous (indicate when caribou are fat)	4
Beach pea, sea pea, wild pea	*Lathyrus japonicus* Willd	Fabaceae	Herb	Edible, avoid	4

^*^These taxa accounted for 430 out of 530 total responses about plant usage, approximately 81% of all responses. Note: Rotten wood is used in the preparation of smoked fish, hence we attribute it to the edible usage category. Taxa are sorted from most reported to least reported

**Table 2:** Table of the 15 taxa reported in two of the three communities that were part of this survey: Postville, Hopedale, and Rigolet.

Reported common name(s)	Most specific classification	Family	Plant Group	Use(s)	Frequency
Poppy	*Papaver nudicaule* L	Papaveraceae	Herb	Garden, decorate	9
Larkspur	*Delphinium* spp*.*	Ranunculaceae	Herb	Garden, decorate	8
Snowberry, whiteberry, fever tea, Maynard tea, maidenhair	*Gaultheria hispidula* (L.) Muhl. ex Bigelow	Ericaceae	Herb	Edible, medicinal, fire, miscellaneous (partridge food)	8
Marshberry	*Vaccinium oxycoccos* L	Ericaceae	Shrub	Edible	6
Dempsum, wild plum, pear tree, prune tree	*Amelanchier bartramiana* (Tausch) M.Roem	Rosaceae	Shrub	Edible, garden, avoid, miscellaneous (animal food)	5
Evergreen	Family Pinaceae	Pinaceae	Tree	Medicine, design, decorate	5
Moss	Division Bryophyta	–	Non–vascular	Medicinal, design	4
Caribou moss	*Cladonia* spp*.*	Cladoniaceae	Lichen	Medicinal, design, miscellaneous (dog food, caribou food)	4
Iris	*Iris setosa* Pall. ex Link	Iridaceae	Herb	Garden	4
Alexander, alexander plant	*Ligusticum scoticum* L	Apiaceae	Herb	Edible	4
Lupine	*Lupinus polyphyllus* Lindl	Fabaceae	Herb	Garden, game, avoid	4
Black spruce, spruce	*Picea mariana* (Mill.) Britton, Sterns & Poggenb	Pinaceae	Tree	Edible, medicinal, fire, design	4
Ground juniper	*Juniperus communis* L	Cupressaceae	Tree	Edible, medicinal, design, miscellaneous (as toilet paper)	3
Thousand leaves, fern, hundred thousand	*Achillea millefolium* L	Asteraceae	Herb	Medicinal, avoid	2
–	*Mertensia maritima* (L.) Gray	Boraginaceae	Herb	Edible, game	2

^*^These taxa accounted for 72 out of 530 total responses about plant usage, approximately 13% of all responses. Taxa are sorted from most reported to least reported

**Fig. 2:**
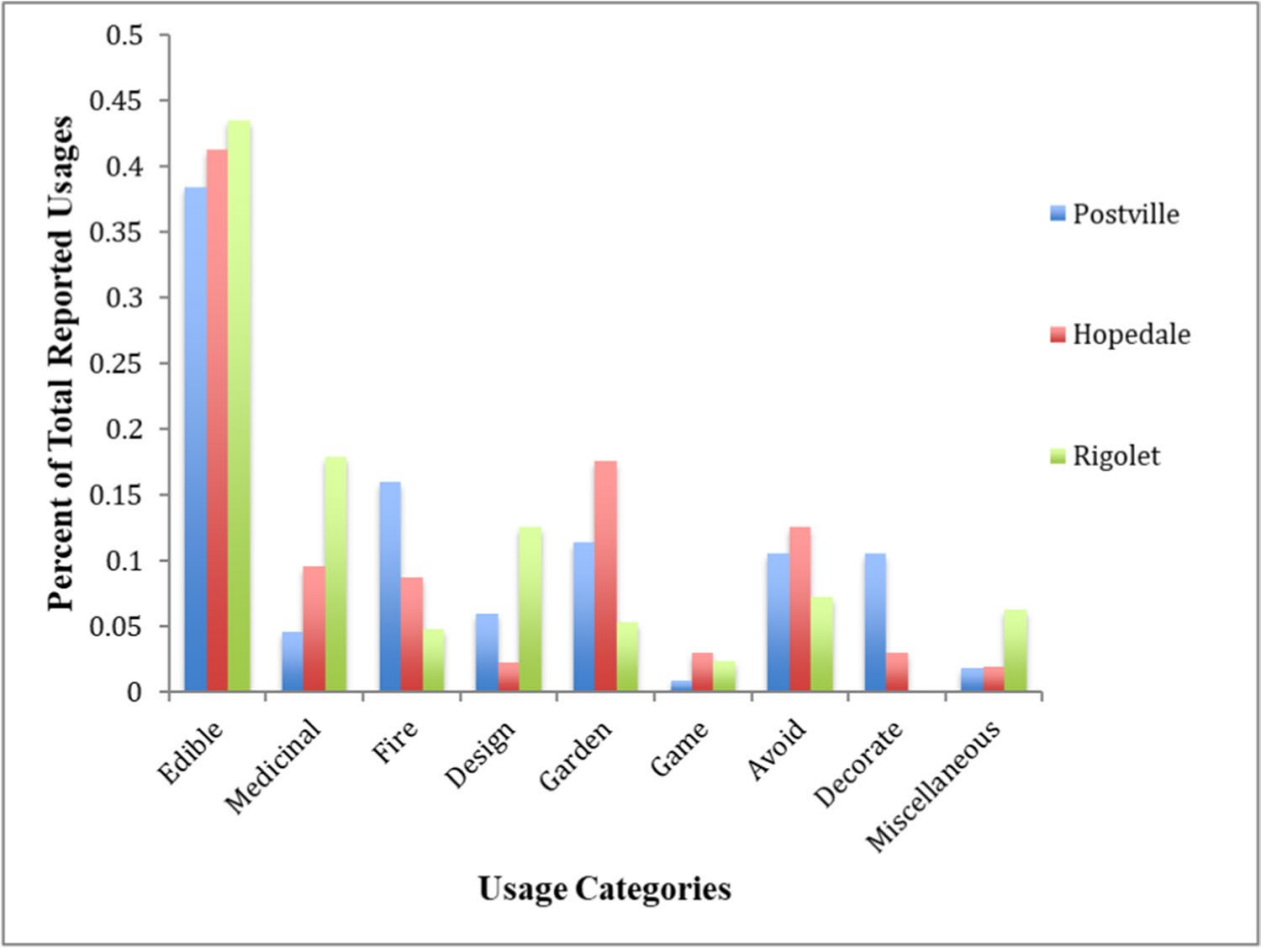
Bar graph illustrating the percent of total reported usages by usage category in Postville, Hopedale, and Rigolet.

The 34 common taxa accounted for 81% (430 of the 530) of the total responses (Table 1). The 15 taxa common to two of the three communities accounted for about 14% (72 of the 530) of the total responses (Table 2). Considered together, taxa common to all three communities and taxa common to two communities made up 95% of all responses (502 of 530). The edible usage category was the most frequent across all three communities (Fig. 2).

**Fig. 3:**
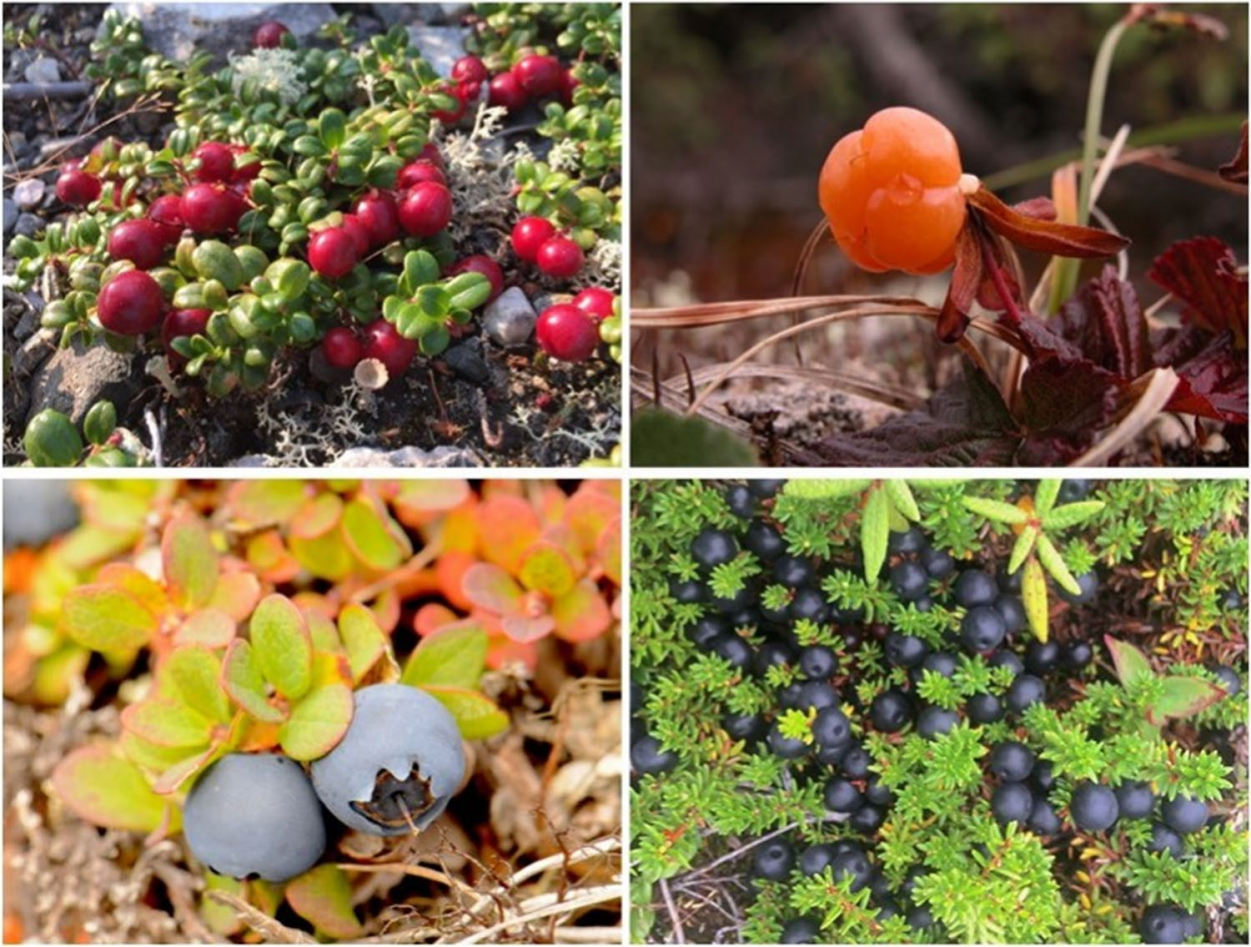
Photo panel of four berry–producing species, from left to right and top to bottom: redberry, bakeapple, blueberry, and blackberry.

Berry–producing taxa made up most of the reported edible taxa, as well as the top four most reported ones (Table 1). Bakeapple (*Rubus chamaemorus*), blueberry (*Vaccinium* spp.), redberry (*Vaccinium vitis–idaea*), and blackberry (*Empetrum nigrum*) were simultaneously the most reported taxa overall and the most reported edible taxa (Fig. 3).

### Selected Examples of Cultural Importance

Berry picking is an important annual cultural activity in northern Labrador, and every person interviewed had something to say about picking berries! One community member said, “Everyone gets their berries!” and that quote succinctly sums up how integral the annual berry harvest is to the cultural calendar in Labrador. In Rigolet, a community member discussed with pride his family’s long–standing tradition of berry picking together. Some berries were held in great esteem, such as the bakeapple with its golden drupelets, which was described as “priceless,” “a priceless gift,” and “[their] gold.” Especially in the case of the bakeapple, people traveled great distances by boat out to islands or inland to find suitable patches. Older community members, finding it difficult to travel, lamented that berry patches closer to town were being ruined by road dust, snowmobile damage, and careless garbage disposal. Picked berries are eaten raw and are often made into baked goods such as squares (a type of cake filled with fruit, cut into small pieces), puddings, cheesecakes, pies, jams, and jellies. Blackberry cake and redberry squares were two often–mentioned recipes, the blackberry cake being fondly remembered as a special treat by older generations.

The smoking of fish was another cultural activity in which plants played an integral role. A community member in Hopedale explained how berry sods are used to smoke fish:

“We call them sods. We don’t call them berry bushes. It goes for redberries. You make a square out of the ground where the redberries grows, make it around 10 inches thick...cut it 16 by 12, need about three or four of them for a smoke, one batch of fish. There are other people that likes blackberries sods, but I like the redberries in general. Some uses birch wood...they get that from out on the land.”

A community member from Postville remembered summers from her childhood where she smoked and salted fish continuously, and the role the plants played in this process:

“He [my father] and the boys would bring in the most fish from the outside...he would dry and pile the fish in the fish shed and salt it so that it wouldn’t go bad. That’s how it was. Smoking fish was another thing, smoking fish continuously all summer. That was food for our table. Smoked it with berry leaves and rotten wood... and then sawdust on top of that so that the... flames wouldn’t come up... sprinkle a little bit of water to keep it from catching. Didn’t want to burn his smokehouse and lose his fish!”

In the case of smoking fish, berry sods, wood, and rotten wood provided the heat and smoke that both dried the fish to preserve, as well as providing a flavor appreciated by many community members.

It became clear that plants served as memory markers for defining historical events in Postville, Hopedale, and Rigolet, such as the history of the Moravians and the relocations that happened in the late 1950s (Dombrowski et al. 2016; Stopp 2009). When people were asked about mushrooms (Division Basidiomycota), rhubarb (*Rheum compactum*), poppies (*Papaver nudicaule*), and chives (*Allium schoenoprasum*), they recalled the history of the Moravian missions and missionaries in northern Labrador. In the case of mushrooms, few people recalled ever picking wild mushrooms, but they remembered that these were a favorite of the Moravians. Relocated Inuit now living in Hopedale expressed memories of their former communities when discussing rhubarb, cottongrass (*Eriophorum* spp.), and wild chives. Trips back to the old settlements like Hebron are important and incredibly emotional, and memories of these reunions were triggered by picking blackberries and eating seaweed (Class Phaeophyceae). A relocatee in Hopedale recalled crying so hard at one of the Hebron reunions while picking blackberries that she accidentally picked up pieces of animal feces and put them into the bucket with the rest of the berries. Another woman, attending a similar reunion at Hebron, remembered that she saw people eating seaweed at that event. Plants marked other important events, too. Non–native species were brought in the communities over the years. The building of the new school in Postville brought in butter and eggs (*Linaria vulgaris*) with the lumber, and vetch (*Vicia cracca*) was introduced to Postville via hay that was brought in years ago for a beloved horse named Queenie. In Hopedale, one woman said pink clover (*Trifolium pratense*) was introduced via the sod used to turf the new playground. When discussing plants, people recalled these historical events, both major and minor, of northern Labrador.

When talking about plants, community members often recalled who taught them about a certain plant, or with whom they associated a specific plant. One woman in Hopedale recalled her father bringing her spruce gum (*Picea* spp.) as a treat when he returned from checking his traps. A man recalled “going wooding” with his father, and how his father gave him a piece of spruce gum to help him breathe and clear up his cold. An elder in Hopedale recalled her mother boiling spruce bows to make a tonic for cleaning the blood. A woman, though she had never tried it herself, remembered her grandmother eating the new alder leaves (*Alnus alnobetula* subsp. *crispa*) and the tops of roseroot (*Rhodiola rosea*). Importantly, discussion about plants also brought up reasons why someone may not have learned as much about plants from their parents and grandparents as they now wish they had. One woman, expressing sadness that she did not know more about plants and their traditional uses said:

“I never used to watch and that’s why I never learned much...I’d just run off. It’s like we didn’t care...we didn’t want to learn or something...and now I regret it...not learning from them. Mostly I did [learn from them], but not the most important things, I suppose.”

Through discussing plants, it was clear that plants were evidence of knowledge transfer between generations, and the respect and status of this knowledge is reflected in the sadness of those who wish they had learned more from their parents and grandparents when they had the chance. Plants are also a means through which people monitor environmental changes and understand ecological relationships. In both Hopedale and Postville, interviewees noted the rapid change over the last few decades concerning the increase in the number and growth rate for willows (*Salix* spp.), alders, and balsam poplar (*Populus balsamifera*). A man in Hopedale said, “something happened to the climate, made them go boom!” Willows, in addition to marking changes in climate, were noted by a few interviewees as a plant used to indicate water on the land. Berries, but particularly bakeapples, are said by locals to be sensitive to too much heat and too much sun. A woman in Postville said that there are now years with no berries at all because it is too hot and dry, and she felt climate change was to blame for this. In Rigolet, a few people recalled fireweed (*Chamaenerion angustifolium*) being called salmon flower because the blooms corresponded with the arrival of the salmon (*Salmo salar*). In Hopedale, an Elder said that appearance of the fluffy heads of cottongrass meant that the backs of the caribou (*Rangifer tarandus*) were full of fat. The amount of fruit set by the mountain ash (*Sorbus decora*) was noted as a predictor for snowfall in the coming winter. Finally, people noted the importance of plants in the diets of animals they hunt. Caribou lichen (*Cladonia* spp.) is a staple of the caribou diet. Snowberries (*Gaultheria hispidula*), redberries, blackberries, spruce buds, and willow seeds are noted as food for partridges (*Lagopus muta* and *L. lagopus*). Plants, in multiple ways, are a medium through which community members understood and monitored the environment around them.

Finally, discussions about plants revealed that plants supported and maintained traditional values and conventions concerning traditional usage of natural resources. Traditional values supported by plants included sharing with others, sustainable usage, and living off the land. Berry species seemed to be particularly important concerning the maintenance and expression of traditional values. In Postville, one woman we interviewed made it clear that when picking berries, you did not pick every day and you did not over pick, and you share with others when you can. Another woman said that berries used to be shared, but now people sell them for high prices, particularly the bakeapples. When talking about harvesting wood for home heating, the same woman also said that you should not use someone else’s wood path, i.e., the trail in the woods they had cut to access firewood, because it would be disrespectful to do so. Across all interviews, it was clear that there was great pride in being on and living off the land and using and being on the land was an integral part of local identity. When discussing gardening, a woman in Hopedale said, “We’ve always lived off the land, and gardening is just another arm of that.” Another woman interviewed, who was also discussing gardening, but instead explaining why not everyone gardens, said, “Our people have always been hunters and gatherers, but our people aren’t croppers.” When discussing plants as medicine, and what seemed to be their decreased use over time, multiple people expressed frustration that they do not use more medicines from the land for the general ailments.

## Discussion

The main goal of this research was to understand the broader cultural value of plants in Hopedale, Postville, and Rigolet, three communities in Nunatsiavut. We found that plant usage is similar among the three communities suggesting a common body of knowledge (Table 1 and Table 2). Secondly, but more importantly, speaking with community members in Postville, Hopedale, and Rigolet shone a light on the integral ways that plants are part of life on the north coast of Labrador.

With the completion of this work, all five communities in Nunatsiavut have been included in contemporary plant use surveys. Clark (2012; Cuerrier et al. 2019) reported 58 taxa in Nain, which is similar to the richness among the three communities discussed in this paper. Concerning usage reported by Clark, the most common reported usage was edibility, and she emphasized that berries were a highlight for edible plants, much like what was found in Postville, Hopedale, and Rigolet. Makkovik has also been the focus of a recent survey by Oberndorfer (2016). Oberndorfer reported 65 taxa, similar richness to this survey. Although not reported in the survey, the most common usage category was edible, and there were 11 berry–producing species reported in the edible usage category. Results presented here are consistent with Oberndorfer’s (2016) work in Makkovik in that edibility was the most common usage with a distinct focus on berries and a similar number of taxa were reported.

Other texts note the value of plant usage to communities in Labrador over the last century. The work of Brice-Bennett et al. (1977) paved the way for the Inuit land claim agreement and the existence of Nunatsiavut as an autonomous territory by showing the intimate connection between the communities on the north coast of Labrador and their environment over millennia. In this powerful text, there is a chapter on Postville that includes a list of berries used by community members, all of which were also documented in this survey, and there is even a map that details the locations of berry patches around Postville. There are examples of berry toponyms given in the book, further testament to the importance of berries—and plants at large—to local communities. In northern Labrador, Hutton (1912) and Peacock (1947), both medical doctors, provided brief notes on plants usage. Hutton noted that berries and willow were eaten, and berries were an especially important food source. Hutton noted only a single example of medicinal plant use, referring to “twigs of rosemary” that were made into a tea and drank for any illness. The twigs to which he refers are most likely Labrador tea (*Rhododendron groenlandicum*) and their usage as a medicinal tea continues today in Postville, Hopedale, and Rigolet. Peacock (1947) noted Labrador tea, willow, roseroot, puffball (Division Basidiomycota), and tamarack (*Larix laricina*; common name in this region being juniper tree) as medicinal taxa, all five of which were noted in this survey as having medicinal uses. Studying country food consumption in Makkovik, Mackey and Orr (1987) found that, in total, surveyed households collect 832 kg of berries, mainly redberry, blackberry, blueberry, bakeapple, and squashberry (*Viburnum edule*). All of the berries were noted as still being used in both a recent survey of Makkovik (Oberndorfer 2016), in addition to the former four taxa being the most reported plants in the results presented in this paper (see frequency, Table 1), a testament to their continued importance to the communities as a valued food source and cultural item.

In Postville, Hopedale, and Rigolet, the depth of the relationships between plants and local culture is undeniable and the complexity of relationships became more integral and complicated, as the layers of culture were understood. Most obvious were the direct uses for plants, and these obvious uses are reflected in other plant use surveys conducted in Nunatsiavut. After direct uses, the ways that plants are linked to cultural activities—like berry picking, smoking fish, and wooding—became understood. These cultural activities, in turn, provide quality of life for community members by providing culturally relevant food sources, i.e., supporting food sovereignty, in addition to heating homes in an environment that would be almost impossible to inhabit without heating. Smoking fish and wooding are noted as integral cultural activities by Clark (2012) and Oberndorfer (2016), and accounts from across the Arctic and Subarctic attest to the widespread importance of berry picking as a cultural activity, both historically and presently (Boulanger-Lapointe et al. 2019; Hawkes 1916; Jones 1983; Murray et al. 2005; Zutter 2009). Inuit have harvested wood for centuries in this area, possibly even shaping the local environment while doing so (Lemus-Lauzon et al. 2012), and we noted the value of wood as a raw material and a source of heat through burning it, as well as the value of the cultural activity of going to collect wood.

The deeper levels of plant–people relationships included plants as memory markers, expressions of ecological awareness, a catalyst for intergenerational knowledge exchange, and a medium to express and encourage traditional values. Plants as markers of local history were noted by Oberndorfer (2016) in Makkovik, particularly poppies and rhubarb as reminders of the Moravians, as they were noted in this study. Examples of plants acting as a means for people to monitor their environment are many, both in Labrador and the larger Arctic and Subarctic. Siegwart Collier (2020) noted that people felt increased tree growth and cover was shading berries and decreasing harvests. Clark (2012) noted certain flowers referred to as bumblebee food in Nain. Other texts from Nunavik noted flowers as bumblebee food or *igutsaup nigingit*, too, in addition to cottongrass (Cuerrier and Elders of Kangiqsualujjuaq 2012; Cuerrier and Elders of Kangirsujuaq 2005; Cuerrier and Elders of Umiujaq and Kuujjuarapik 2011). Joamie and Ziegler (2009), and Mallory and Aiken (2012) found that mountain avens (*Dryas integrifolia*) can be used to judge the season, and thus predict when it is to time for certain seasonal activities, and, again, Oberndorfer (2016) found that the ripening of blackberries was linked to the arrival of the geese in the fall. She also noted that people linked the blooming of pond lilies (*Nuphar* spp.) to the ripening of bakeapples, another example of plants acting as expressions of ecological awareness. Plants as a catalyst and medium for intergenerational knowledge exchange was noted by Joamie and Ziegler (2009), when describing learning about plants from parents, and reports about tree usage in Nain referred to learning from family members (Lemus-Lauzon et al. 2012).

The final point about the importance of plants in expressing and continuing traditional cultural values is perhaps the deepest layer of plant–people relationships understood from this survey, and is also perhaps the most difficult to locate in other texts. A presentation at the 41st meeting of the Society of Ethnobiology by Elder Annie Evans (2018) from Makkovik, Nunatsiavut, discussed how plants are linked to customary laws governing the usage of natural resources, such as sharing resources and respecting the land, and such customary laws are an integral aspect of local identity in Nunatsiavut (Brice-Bennett et al. 1977). Being on the land and living off the land is a cultural foundation in Nunatsiavut, as it also is in other communities both in and outside of the north (Greenwood and de Leeuw 2007; Ohmagari and Berkes 1997; Oster et al. 2014). The collection and distribution of plant resources (such as berries) is a means to practice values such as sharing, being on the land, and living off the land, without degrading it.

## Conclusion

We finish this project looking forward. We did not consider gendered dimensions of plant use in our survey, but that could be an interesting aspect of a future project. The completion of this project means that all five communities in Nunatsiavut have participated in recent ethnobotanical surveys: Nain in Clark (2012), Makkovik in Oberndorfer (2016), and Postville, Hopedale, and Rigolet in this work presented here. Our main focus for future work in this area will be bringing together all of the recent ethnobotanical surveys in Nunatsiavut. We aim to unite these surveys in a text for use by the communities that celebrates the rich, culturally integral plant knowledge across Nunatsiavut. We envisage something similar to the plant use booklet we helped produce in Nain (Downing et al. 2012), with illustrations and interview excerpts that showcase the immense cultural value of these plants in Nunatsiavut.

Plants are an integral part of life in Postville, Hopedale, and Rigolet. Reported plants almost totally overlapped among the three communities, suggesting a shared body of culturally rooted plant knowledge, perhaps due to movement among communities and family relations in other communities, both of which were mentioned in interviews. From their direct application in cultural practices—such as smoking fish, berry picking, and wooding—to the fundamental ways that they support local memory, knowledge exchange, ecological awareness, and traditional values, plants continue to be an integral part of life and culture in Nunatsiavut.

## Supplementary Information

Below is the link to the electronic supplementary material.

Supplementary file1 (XLSX 30 kb)
